# Association of inbreeding and regional equine leucocyte antigen homozygosity with the prevalence of insect bite hypersensitivity in Old Kladruber horse

**DOI:** 10.1111/age.13075

**Published:** 2021-05-10

**Authors:** L. Vostry, H. Vostra‐Vydrova, J. Citek, G. Gorjanc, I. Curik

**Affiliations:** ^1^ Czech University of Life Sciences Kamycka 129 Prague 16500 Czech Republic; ^2^ Institute of Animal Science Pratelstvi 815 Prague 10400 Czech Republic; ^3^ South Bohemia University Branisovska 31a Ceske Budejovice 370 05 Czech Republic; ^4^ Veterinary Research Institute Hudcova 296/70 Brno 621 00 Czech Republic; ^5^ The Roslin Institute and Royal (Dick) School of Veterinary Studies University of Edinburgh, Easter Bush Edinburgh Midlothian EH259RG UK; ^6^ Faculty of Agriculture University of Zagreb Svetošimunska cesta 25 Zagreb 10000 Croatia

**Keywords:** genomics, horse, inbreeding, inbreeding depression, insect bite hypersensitivity, pedigree

## Abstract

Inbreeding depression is the reduction of performance caused by mating of close relatives. In livestock populations, inbreeding depression has been traditionally estimated by regression of phenotypes on pedigree inbreeding coefficients. This estimation can be improved by utilising genomic inbreeding coefficients. Here we estimate inbreeding depression for insect bite hypersensitivity (IBH) prevalence, the most common allergic horse disease worldwide, in Old Kladruber horse. In a deep pedigree with 3214 horses (187 genotyped), we used a generalised linear mixed model with IBH phenotype from 558 horses examined between 1996 and 2009 (1368 records). In addition to the classical pedigree information, we used the single‐step approach that enabled joint use of pedigree and genomic information to estimate inbreeding depression overall genome and equine leucocyte antigen (*ELA*) *class II* region. Significant inbreeding depression was observed in all models fitting overall inbreeding coefficients (odds ratio between 1.018 and 1.074, *P* < 0.05) with the exception of Kalinowski’s new inbreeding (*P* = 0.0516). The increase of *ELA class II* inbreeding was significantly associated with increased prevalence of IBH (odds ratio 1.018; *P* = 0.027). However, when fitted jointly with the overall inbreeding coefficient, the effect of *ELA class II* inbreeding was not significant (odds ratio 1.016; *P* = 0.062). Overall, the higher *ELA class II* and/or overall inbreeding (pedigree or genomic) was associated with increased prevalence of IBH in Old Kladruber horses. The single‐step approach provides an efficient use of all the available pedigree, genomic, and phenotype information for estimation of overall and regional inbreeding effects.

Mating of relatives is unavoidable in closed and finite populations and can lead to inbreeding depression – higher incidence of defects caused by recessive alleles and redistribution of genetic variance within and between populations. Inbreeding depression is a well‐documented phenomenon, known as the reduction of performance in quantitative traits under constant selection pressure such as fitness and health traits (Crnokrak & Roff 1999; Bittles & Black 2010; Leroy 2014).

In livestock populations, inbreeding depression has been traditionally estimated by regression of phenotypes on pedigree inbreeding coefficients (Baker *et al*. 1945; Leroy 2014). Alternatively, some other pedigree‐derived coefficients, for example Kalinowski’s new inbreeding coefficient, have been successfully used (Mc Parland *et al*. 2009; Doekes *et al*. 2019; Curik *et al*. 2020). Development of large scale high‐throughput genotyping has enabled quantification of individual genomic inbreeding in human (McQuillan *et al*. 2008), wild (Santure *et al*. 2010), and livestock populations (VanRaden 2008; Curik *et al*. 2014). The substitution of the pedigree with genomic inbreeding coefficient, improves the estimation of inbreeding depression from several perspectives (Kardos *et al*. 2016; Curik *et al*. 2017). In livestock populations, there are often many individuals in the pedigree that have phenotype records but are not genotyped. Legarra *et al*. (2020) showed how to estimate inbreeding coefficients by combining pedigree and genomic relationship matrices, which is commonly referred to as the single‐step approach (Legarra *et al*. 2009). Recently, this approach was used in the estimation of inbreeding depression for semen traits in the Basco‐Béarnaise dairy sheep breed (Antonios *et al*. 2021).

Classical horse breeds are often bred as closed populations and are known to reach high inbreeding often accompanied by inbreeding depression. Consequently, inbreeding depression has been observed for racing performance (Klemetsdal 1998; Todd *et al*. 2018), reproductive traits (Van Eldik *et al*. 2006; Müller‐Unterberg *et al*. 2017), morphological traits (Gómez *et al*. 2009; Vostrý *et al*. 2011), and health traits (Sánchez‐Guerrero *et al*. 2019).

The Old Kladruber horse, an old Czech breed established in 1579 that belongs to the Spanish horse group, has been maintained in a closed population for a long time with recent average inbreeding of 0.13 (Vostrá‐Vydrová *et al*. 2016). In this population, inbreeding depression has been already observed for melanoma and vitiligo grading (Hofmanová *et al*. 2016) as well as for reproductive traits (Šichtař *et al*. 2017). The breed is affected also by insect bite hypersensitivity (IBH), the most common allergic skin disease caused by the bites of insects of the genus *Culicoides*. There is a common consensus that IBH is genetically controlled, and the starting impulse is a bite by midges of the *Culicoides* spp. and less frequently *Simulium* spp. The subsequent allergic reaction is a result of hypersensitivity to the allergens contained in their saliva (van der Rijt *et al*. 2008). The main symptoms are pruritus (itchiness), scaling, and hair loss. Clinical manifestation of IBH is due to an allergic reaction sharing some similarities with human atopic dermatitis. IBH is appearing in various horse breeds all over the world as a complex disease affected by environmental and genetic factors (Unkel *et al*. 1987; Eriksson *et al*. 2008; Schurink *et al*. 2011).

The impact of inbreeding on the IBH was analysed in Dutch Friesian horse and found non‐significant (Schurink *et al*. 2011). In contrast, Andersson *et al*. (2012) have shown that increased homozygosity across the entire equine leucocyte antigen (*ELA*) *class II region* is associated with a higher risk of IBH. Unfortunately, Andersson *et al*. (2012) did not analyse if overall or regional inbreeding increases the risk of higher IBH.

The aim of this study was to estimate the effects of inbreeding on the prevalence of IBH in the Old Kladruber horse by logistic linear mixed regression from data with a heterogeneous combination of pedigree, genomic, and phenotypic information. Inbreeding was based on the combined pedigree–genomic single‐step approach of Legarra *et al*. (2009, 2020) and compared with the classic pedigree approach. We also estimated the effect of *ELA class II* regional inbreeding to test the results from Andersson *et al*. (2012). With respect to the prevalent heterogeneous combination of pedigree, genomic, and phenotypic information in many livestock populations, the application of single‐step approach is empowering.

## Materials and methods

### Phenotype, pedigree, and genomic information

**Figure 1:**
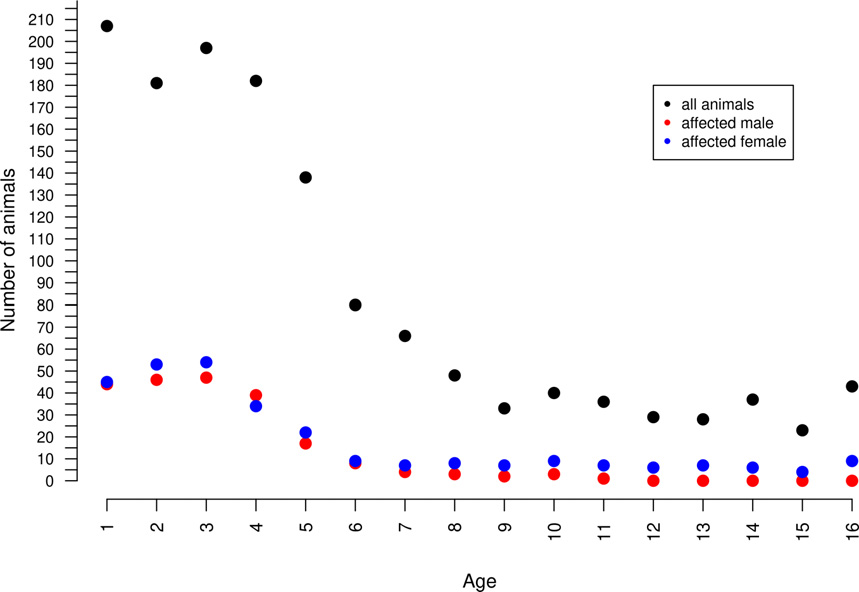
The number of animals with insect bite hypersensitivity by sex and age at the inspection.

The analysis was based on 1368 IBH inspections from 558 horses. We assumed that all horses have experienced the same probability of mosquito bites as all phenotyped horses have been kept at the same stud and were exposed to the same environmental factors, including biting insects. The National Stud Kladrub is situated in a temperate climate zone, at an altitude of about 200 m above the sea level in the eastern part of Bohemia, Czech Republic. While conditions are favourable for horses, they are also favourable for biting insects such as *Culicoides*, for example *Culicoides* *nubeculosus* and *Culicoides* *obsoletus* (van der Rijit *et al*. 2008). The data on the Old Kladruber horses were provided by the stud book. The inspections were performed visually twice a year (May and October) between 1996 and 2009 by the same person on a binary scale with respect to the prevalence of the typical skin lesions (0 when clinical symptoms were absent and 1 when clinical symptoms were present). Similar IBH scoring system was also used by Grevenhof *et al*. (2007), Peeters *et al*. (2015) and Schurink *et al*. (2011). During the measurement period, 558 horses were inspected several times and 29% of horses (163) showed clinical symptoms of IBH. The pattern of the insect bite hypersensitivity prevalence with respect to the sex and horse age at the inspection is presented in Fig. 1.

This prevalence was much higher compared to 8.1% observed in Swedish‐born Icelandic horses (Eriksson *et al*. 2008), 8.8% in Shetland ponies (Schurink *et al*. 2009), 18.2% in Dutch Friesian broodmares (Schurink *et al*. 2011), and 10% in Belgian Warmblood horses (Peeters *et al*. 2015).

The total pedigree was up to 52 generations deep and comprised of 3214 individuals, with mean complete generation equivalent (EQC, Maignel *et al*. 1996) equal to 15.58 for the inspected horses. Overall, 187 horses were genotyped with the Equine SNP70 BeadChip (Illumina), consisting of 65 102 evenly distributed SNPs over the equine genome. This genotyping has been performed as a part of the routine diversity management, recently introduced into the breeding programme.

SNP data were edited and analysed using plink 1.9 (Chang *et al*. 2015). Only autosomal SNPs with known chromosomal position were analysed with individual call rate >0.9 and SNP call rate >0.9. The minor allele frequency (MAF) was filtered according to two different approaches. Filtering for MAF >0.01 was performed in the calculation of the ***G*** matrix and FG, while filtering for MAF was not performed in the calculation of runs of homozygosity (ROH‐based genomic inbreeding coefficients as recommended by Meyermans *et al*. (2020). To define the parameters of an ROH, we set the minimum density to 0.05 Mb/SNP and the largest gap to 1 Mb. The minimal number of SNPs in each ROH was set to 20. Similar parameters were used by Todd *et al*. (2018). The maximum number of heterozygous SNPs allowed within ROH >5 Mb was 0 and maximum number of missing SNPs allowed within ROH >5 Mb was 1. Unfortunately, only 47 inspected horses were genotyped (seven were affected and 40 were unaffected by IBH) while the remainder of 137 horses were not phenotyped (inspected for IBH). However, all included horses were highly related and were kept in the same environment, on the National Stud in Kladruby nad Labem.

### Estimation of pedigree and genomic inbreeding coefficients

We estimated three different pedigree inbreeding coefficients: classical (*F*_X_); classical restricted to five generations (*F*_X5_); and Kalinowski’s new (*F*_NEW_) inbreeding coefficient. When pedigree depth varies considerably, estimation of inbreeding depression can be biased as individual inbreeding coefficient estimates refer to different base populations. To avoid this potential bias, we used the *F*_X5_ as all phenotyped horses had complete pedigrees for at least five generations. Note that this inbreeding coefficient refers to the recent inbreeding. *F*_NEW_ is defined as the probability that any allele in an individual is autozygous for the first time and is assumed to better capture inbreeding depression caused by alleles with large detrimental effects or detrimental alleles that have not been purged (Doekes *et al*. 2019; Curik *et al*. 2020). *F*_X_ and *F*_X5_ were calculated by PEDIG software (Boichard 2002), while *F*_NEW_ (Kalinowski *et al*. 2000) was calculated by gene dropping with 1 000 000 repetitions as suggested by (Baumung *et al*. 2015) and with corrections implemented in the grain 2.2 software (Doekes *et al*. 2020).

Following the idea presented in Legarra *et al*. (2020) we estimated genomic inbreeding coefficients for all horses, by combining pedigree and genomic information with the single‐step approach. This approach projects genomic relationships (***G*** matrix; VanRaden 2008) to all ungenotyped individuals in a pedigree (***A*** matrix; Legarra *et al*. 2009; Christensen & Lund 2010). The resulting projected matrix is often denoted as the ***H*** matrix. The projection requires careful consideration to account for different base populations of pedigree and genomic information. To address this, we used one ‘meta‐founder’ for pedigree and expected allele frequency equal to 0.5 (Legarra *et al*. 2015). With this approach, we estimated three genomic inbreeding coefficients as the diagonal of ***H*** matrix minus one: (a) *F*_H_ representing whole genome; (b) *F*_ELA‐H_ representing the *ELA class II* region with 87 SNPs between 32 and 34 Mb on *ECA20* (Brinkmeyer‐Langford *et al*. 2013); and (c) *F*_noELA‐H_ representing whole genome without the *ELA class II* region. *F*_noELA‐H_ (whole genome SNPs without 87 SNPs covering the *ELA class II* region) was calculated to reduce confounding between overall (*F*_H_) and regional (*F*_ELA‐H_) inbreeding.

In addition, for the genotyped animals we also calculated overall and regional ROH inbreeding coefficient (McQuillan *et al*. 2008; Curik *et al*. 2014), to enable comparison of the inbreeding level in other horse populations. Calculated ROH inbreeding coefficient (*F*_ROH>5Mb_) was defined as the proportion of the genome in ROH of a certain minimal length relative to the whole autosomal genome (*F*_ROH_ = *L*_ROH_/*L*_AUTOSOME_). *ELA class II* ROH inbreeding coefficient (*F*_ELA‐ROH>5Mb_) was defined in the same way but only for the *ELA class II* region. We estimated all genomic inbreeding coefficients with the r software, except for ROH inbreeding coefficients that were estimated with plink 1.9 (Chang *et al*. 2015) following settings of Todd *et al*. (2018).

Note that genomic inbreeding coefficients derived from ***H*** are defined relative to a population with allele frequency of 0.5 for all loci, due to the meta‐founder single‐step approach (Legarra *et al*. 2015). At this allele frequency, we expect 50% heterozygous loci. Therefore, these inbreeding coefficients express deviations from this expectation and an individual with less (more) than 50% heterozygous loci will have positive (negative) inbreeding coefficient. In contrast, pedigree and ROH based inbreeding coefficients are defined relative to a past base population (founders in the pedigree or conceptual base population defined by the ROH length). Therefore, these inbreeding coefficients are expressed as probabilities and strictly between 0 and 1.

### Modelling of inbreeding depression

Depending on the information source used in the estimation of inbreeding coefficients, three different modelling approaches were used. Models from the first approach were based on the pedigree inbreeding coefficients (*F*_X_, *F*_X5_ and *F*_NEW_). In the second approach, three models were based on inbreeding coefficients derived from the single‐step (*F*_H_, *F*_ELA‐H_, and *F*_ELA‐H_ with *F*_noELA‐H_).

Calculations were performed with the GLIMIX procedure, which fitted logistic linear mixed model for repeated measurements (SAS Inst. Inc.). Here, the dependent variable (*y_i_*) has value of 1 (occurrence of IBH clinical symptoms) with probability *π_i_* or 0 (absence of IBH clinical symptoms) with probability 1 − *π_i_* for observation *i*. We modelled this probability as described in Citek *et al*. (2017) with the addition of inbreeding coefficients as covariates:

$$\log\left(\frac{\pi_{\mathrm{ijkl}}}{1‐\pi_{\mathrm{ijkl}}}\right)={\mathrm{Year}}_{i}+{\mathrm{Age}}_{j}+{\mathrm{Sex}}_{k}+bF+p_{l}+a_{l},$$

where Year*_i_* was a fixed effect of the *i*th year of inspection (*i* = 1996, … , 2009), Age*_j_* is a fixed effect of the *j*th year of age of the horse (*j* = 1, … , 16), Sex*_k_* is a fixed effect of the *k*th sex (*k* = stallion or mare; geldings were not included), *pe_l_* is a random permanent environment of the *l*th animal with $p∼N\left(0,I_{p}\sigma_{p}^{2}\right)$, ***I***_p_ is the identity matrix of the appropriate dimensions and $\sigma_{p}^{2}$ is the permanent environmental variance, *a_l_* is a random additive genetic effect of the *l*th animal with $a∼N\left(0,Q\sigma_{a}^{2}\right)$, ***Q*** is one of the following: pedigree relationship matrix ***A*** (Henderson 1976) when pedigree inbreeding coefficients were fitted; or single‐step relationship ***H*** matrix (Legarra *et al*. 2009, 2015) when single‐step inbreeding coefficients were fitted, while *b* presents regression coefficient for the various inbreeding coefficients. Separate models were fitted for each inbreeding coefficient (*F*_X_, *F*_X5_, *F*_NEW_, *F*_H_, and *F*_ELA‐H_). We also modelled joint effects of the *ELA class II* regional inbreeding and overall inbreeding (*F*_H_ with *F*_noELA‐_). The suitability of fixed and random effects included in the model was discussed in Citek *et al*. (2017).

The goodness of fit of each of the analysed models was tested by a second‐order bias correction of Akaike information criterion (AICc), which is corrected based on samples size (Burnham & Anderson 2002):

$$\text{AICc}=‐2\;\log\;\text{likelihood}+2k\left(k+1\right)/\left(n‐k‐1\right),$$

where *k* is the number of estimated parameters and *n* is the number of observations.

## Results and discussion

### Inbreeding level

Descriptive statistics of various inbreeding coefficients, equivalent complete generations, and inspected horses for IBH are presented in Table 1. The observed pedigree inbreeding level in the Old Kladruber horse (0.139), with increase of inbreeding equal to 0.009 per EQC, was higher than inbreeding level, ranging from 0.103 to 0.125, estimated in other horse breeds with very deep and complete pedigrees such as Thoroughbred (Mahon & Cunningham 1982), Lipizzan (Curik *et al*. 2003), Andalusian (Valera *et al*. 2005), and Lusitano (Vicente *et al*. 2012). Somewhat lower inbreeding, but with higher increase in inbreeding level per EQC (0.0134), was observed for the recent (*F*_X5_) as well as for the new inbreeding coefficient (*F*_NEW_; Table 1. Results reveal that the genetic diversity was affected by bottleneck that occurred in the first half of the 20th Century. At that time, there was a substantial reduction in the size of Old Kladruber population. After this period, from 1973, the regeneration process of the Old Kladruber breed began (Vostrá‐Vydrová *et al*. 2016).

**Table 1:** Descriptive statistics for pedigree (*F*_X_, *F*_X5_, *F*_NEW_), genomic (*F*_G_, *F*_ROH>5Mb_, *F*_H_) and ELA class II regional (*F*_ELA‐G_, *F*_ELA‐ROH>5Mb_, *F*_ELA‐H_) inbreeding coefficients, equivalent complete generations (EQC), and horse age at inspection in Old Kladruber Horse.

Variable	Horses	Mean	Standard deviation	Minimum	Maximum
*F*_X_	558	0.139	0.040	0.017	0.252
*F*_X5_	558	0.067	0.030	0.000	0.150
*F*_NEW_	558	0.029	0.011	0.003	0.080
*F*_H_	558	0.399	0.032	0.287	0.485
*F*_ELA‐H_	558	0.343	0.104	0.060	1.087
*F*_ROH>5Mb_	187	0.099	0.037	0.010	0.205
*F*_ELA‐ROH>5Mb_	187	0.051	0.182	0.000	1.000
EQC	558	15.580	0.761	13.010	17.070
Age (years)	558	5.307	4.110	1.000	16.000

F_X_ and F_X5_ ‐ pedigree inbreeding coefficients based on all and five‐generations; F_NEW_ ‐ Kalinowski’s new inbreeding coefficient; F_H_ and F_ELA‐H_ genomic inbreeding coefficients representing whole genome and the ELA class II region; F_ROH_ _>5Mb_ and F_ELA‐ROH>5Mb_ ‐ runs of homozygosity (ROH) inbreeding coefficients calculated from the sum of all ROH segments greater than 5 Mb representing whole genome and the ELA class II region; EQC ‐ equivalent complete generations; Age ‐ year of age of the horse.

Genomic ROH inbreeding level (*F*_ROH>5Mb_) was lower than the estimated pedigree inbreeding level (Table 1) but with comparable increase of inbreeding per generations (0.010) assuming that 5 Mb long ROH estimates are expected to descend from ancestors that are 10 generations back in the pedigree. The genomic ROH inbreeding was higher than observed by in Grilz‐Seger *et al*. (2019) in Lipizzan (0.035), Austrian Hafflinger (0.021), Italian Hafflinger (0.051), and Noriker (0.029) horse breeds but similar to the Bosnian Mountain horse (0.101) observed by Druml *et al*. (2018).

A much higher inbreeding estimate, 0.399 (*F*_H_), was obtained from the relationship matrix ***H*** due to a distant (ancient) base population. There was no notable difference between overall estimates with or without 87 SNPs from the *ELA class II* region as the region is only a small proportion of the genome (results not shown). All estimates of the *ELA class II* regional inbreeding were lower than overall inbreeding estimates (*F*_ELA‐H_ = 0.343 vs. *F*_H_ = 0.399, and *F*_ELA‐ROH>5Mb_ = 0.051 vs. *F*_ROH>5Mb_ = 0.099), which might be a consequence of the heterozygote advantage (overdominance) in the *ELA class II region*.

### Relationship among inbreeding coefficients

**Figure 2:**
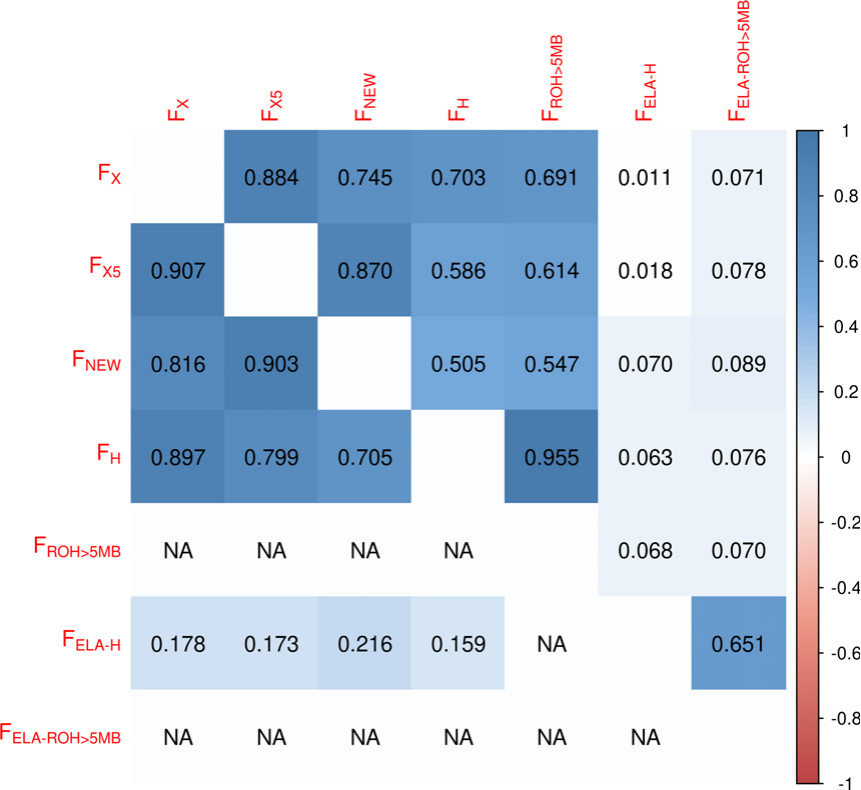
Pearson correlations among pedigree (*F*_X_, *F*_X5_, and *F*_NEW_), genomic (*F*_ROH>5Mb_ and *F*_H_) and ELA class II regional (*F*_ELA‐ROH>5Mb_ and *F*_ELA‐H_) inbreeding coefficients in Old Kladruber Horse. Estimates obtained on 187 (558) genotyped horses are presented in the upper (lower) diagonal. NA marks values that could not be calculated.

Correlations between the various inbreeding coefficients are presented in Fig. 2. High correlations, ranging from 0.505 to 0.955, were observed among all overall inbreeding coefficients (*F*_X_, *F*_X5_, *F*_NEW_, *F*_H_, and *F*_ROH>5Mb_). Low correlations were observed between all overall and regional *ELA class II* inbreeding coefficients, while relatively high correlation was observed between two regional estimates *F*_H‐ELA_ and *F*_ELA‐ROH>5Mb_ (0.651).

### Inbreeding depression

The results of the inbreeding depression analyses for the prevalence of IBH in Old Kladruber Horse are presented in Table 2. Significant overall inbreeding depression, that is the increase of overall inbreeding increased the prevalence of IBH, was estimated in models with *F*_X_, *F*_X5_, and *F*_H_, while the estimate with *F*_NEW_ was in the same direction and close to significant (*P* = 0.0516).

**Table 2:** Inbreeding depression for the insect bite hypersensitivity prevalence in Old Kladruber Horse estimated with the pedigree and single‐step models fitted to the 558 phenotyped horses.

Inbreeding coefficient	Estimate (SE)	Odds ratio	*P*‐value	AICc (ΔAICc)	EEM^1^	Matrix^2^
*F*_X5_	0.071 (0.025)	1.074	**0.0049**	5541 (222)	Table S1	**A**
*F*_X_	0.042 (0.019)	1.043	**0.0277**	5546 (227)	Table S2	**A**
*F*_NEW_	0.138 (0.071)	1.148	0.0516	5551 (232)	Table S3	**A**
*F*_ELA‐H_	0.018 (0.008)	1.018	**0.0270**	5319 (0)	Table S4	**H**
*F*_ELA‐H_	0.016 (0.008)	1.016	0.0620	5361 (42)	Table S5	**H**
*F*_noELA‐H_	0.061 (0.029)	1.063	**0.0365**			
*F*_H_ ^3^	0.068 (0.029)	1.071	**0.0190**	5368 (49)	Table S6	**H**

AICc, corrected Akaike information criterion.

^1^ EEM gives a detailed presentation of models with all estimated environmental effects in the Supported Information (from Tables S1–S6).

^2^ Relationship matrix used; ***A*** is a pedigree relationship matrix (Henderson 1976) and ***H*** is a single‐step relationship matrix that combines all the available genomic and pedigree information (Legarra *et al*. 2009, 2015).

^3^ *F*_H_ is inbreeding coefficient for the whole genome.

F_X_ and F_X5_ ‐ pedigree inbreeding coefficients based on all and five‐generations; F_NEW_ ‐ Kalinowski’s new inbreeding coefficient; F_H_, F_ELA‐H_ and F_noELA‐H_ genomic inbreeding coefficients representing whole genome, the ELA class II and whole genome without the ELA class II region.

When fitted alone in the model, *ELA class II* inbreeding was significantly associated with increasing the prevalence of IBH, corroborating the negative impact of inbreeding on IBH. Interestingly, according to AICc values, the model with *F*_ELA‐H_ as a single covariate was the best model in this study. All models based on the single‐step inbreeding fitted the data better than models based on pedigree inbreeding (Table 2) pointing to the advantage of utilising genomic information in addition to pedigree information.

The presence of a polygenic effect on IBH has been observed in several studies – narrow sense heritability (*h* ^2^) on an IBH status (four categories) was estimated at 0.33 in Swedish‐born Icelandic horses (Eriksson *et al*. 2008) and at 0.16 on a liability scale in Dutch Friesian Broodmares (Schurink *et al*. 2011), between 0.08 and 0.24 in the Dutch Shetland breeding mares (Schurink *et al*. 2009) and between 0.36 and 0.63 in the Old Kladruber horse (Citek *et al*. 2017). The wide range in the last two studies was due to different model definitions. The polygenic nature of IBH was also manifested in several GWAS studies, while several candidate genes located on *ECA7*, *9*, *11* and *20* were also reported (Schurink *et al*. 2012, 2013; Shrestha *et al*. 2015; Velie *et al*. 2016). The most consistent association with IBH across various breeds was observed for the major histocompatibility complex located on *ECA20*, or more precisely for the *ELA* *class I and II* regions (Marti *et al*. 1992; Andersson *et al*. 2012; Schurink *et al*. 2012).

In contrast to other genetic analyses of IBH, this is the first study where significant overall inbreeding depression is reported for IBH. Up to now, the effect of inbreeding on IBH has been estimated only in Dutch Friesian horse (Schurink *et al*. 2011), but no significant estimates were observed. Our results support the positive association of the *ELA class II* region homozygosity with IBH previously reported by Andersson *et al*. (2012). In the models where the overall and regional *ELA class II* inbreeding were analysed simultaneously, regional inbreeding was not significant while overall inbreeding was significant. Still, we think that in the Old Kladruber horse, both overall and *ELA class II* inbreeding are associated with increased prevalence of IBH. We have five arguments this. First, in almost all models overall inbreeding depression was significant, while regional inbreeding depression was significant when fitted independently and gave the best model fit according to AICc. Second, estimates obtained with the single inbreeding covariate models were similar to estimates obtained with two inbreeding covariate models. Third, correlations between overall and regional *ELA class II* inbreeding coefficients were very small, which should exclude potential confounding. Fourth, the regional inbreeding depression was close to significant (*P* = 0.062) in the two inbreeding covariate model. Fifth, significant regional inbreeding depression was observed in other studies (Andersson *et al*. 2012).

Overall, models based on the single‐step relationship matrix combining genomic and pedigree information outperformed models based only on pedigree information (Table 2). The utilisation of all available information in the estimation of inbreeding depression seems intuitively logical, although computer simulations are required to better explore all properties and assumptions of the applied models. With a recent estimate of inbreeding depression for semen traits in the Basco‐Béarnaise dairy sheep breed (Antonios *et al*. 2021), this is the first study to use the single‐step approach to estimate total and regional inbreeding depression. This option might be important for analysing traits where genes with large detrimental impact are participating in the inbreeding depression, particularly as the regional inbreeding might substantially differ from the expected inbreeding estimated by the pedigree inbreeding coefficients (Kardos *et al*. 2016; Curik *et al*. 2017).

## Conclusions

The overall pedigree or genomic and regional *ELA class II* inbreeding were positively associated with the prevalence of insect bite hypersensitivity in Old Kladruber horses. The utilisation of single‐step inbreeding coefficients that combines all the available genomic and pedigree information is a useful approach in the estimation of detrimental inbreeding effects.

## Supporting information

**Table S1**. Estimated environmental and inbreeding effects with significant impact on insect bite hypersensitivity prevalence in Old Kladruber Horse: model is based on pedigree relationship matrix and five generations inbreeding coefficient (*F*_X5_); model AICc value was equal to 5541.
**Table S2**. Estimated environmental and inbreeding effects with significant impact on insect bite hypersensitivity prevalence in Old Kladruber Horse: model is based on pedigree relationship matrix and classical inbreeding coefficient (*F*_X_); model AICc value was equal to 5546.
**Table S3**. Estimated environmental and inbreeding effects with significant impact on insect bite hypersensitivity prevalence in Old Kladruber Horse: models is based on pedigree relationship matrix and Kalinowski’s new inbreeding coefficient (*F*_NEW_); model AICc value was equal to 5551.
**Table S4**. Estimated environmental and inbreeding effects with significant impact on insect bite hypersensitivity prevalence in Old Kladruber Horse: models is based on single‐step relationship matrix and inbreeding coefficient representing the *ELA class II* region (*F*_ELA‐H_); model AICc value was equal to 5319.
**Table S5**. Estimated environmental and inbreeding effects with significant impact on insect bite hypersensitivity prevalence in Old Kladruber Horse: model is based on single‐step relationship matrix and inbreeding coefficients representing the *ELA class II* region (*F*_noELA‐H_) and the whole genome without the *ELA class II* region (*F*_noELA‐H_); model AICc value was equal to 5361.
**Table S6**. Estimated environmental and inbreeding effects with significant impact on insect bite hypersensitivity prevalence in Old Kladruber Horse: model is based on single‐step relationship matrix and inbreeding coefficient representing the whole genome (*F*_ELA‐H_); model AICc value was equal to 5368.

## Data Availability

Phenotypic and genotypic data were obtained from National Stud in Kladruby nad Labem. These datasets were used under a material transfer agreement, and hence, are not publicly available. However, data are available upon request to LV and with the permission of National Stud in Kladruby nad Labem.
