# Supplementary material for: Association of inbreeding and regional equine leucocyte antigen homozygosity with the prevalence of insect bite hypersensitivity in Old Kladruber horse
Source: Anim Genet. 2021 May 10;52(4):422–30. doi: 10.1111/age.13075 (PMC8360196; doi:10.1111/age.13075)
Supplement: Supplementary file 1 — Table S1. Estimated environmental and inbreeding effects with significant impact on insect bite hypersensitivity prevalence in Old Kladruber Horse: model is based on pedigree relationship matrix and five generations inbreeding coefficient (FX5); model AICc value was equal to 5541. Table S2. Estimated environmental and inbreeding effects with significant impact on insect bite hypersensitivity prevalence in Old Kladruber Horse: model is based on pedigree relationship matrix and classical inbreeding coefficient (FX); model AICc value was equal to 5546. Table S3. Estimated environmental and inbreeding effects with significant impact on insect bite hypersensitivity prevalence in Old Kladruber Horse: models is based on pedigree relationship matrix and Kalinowski’s new inbreeding coefficient (F NEW); model AICc value was equal to 5551. Table S4. Estimated environmental and inbreeding effects with significant impact on insect bite hypersensitivity prevalence in Old Kladruber Horse: models is based on single‐step relationship matrix and inbreeding coefficient representing the ELA class II region (F ELA‐H); model AICc value was equal to 5319. Table S5. Estimated environmental and inbreeding effects with significant impact on insect bite hypersensitivity prevalence in Old Kladruber Horse: model is based on single‐step relationship matrix and inbreeding coefficients representing the ELA class II region (F noELA‐H) and the whole genome without the ELA class II region (F noELA‐H); model AICc value was equal to 5361. Table S6. Estimated environmental and inbreeding effects with significant impact on insect bite hypersensitivity prevalence in Old Kladruber Horse: model is based on single‐step relationship matrix and inbreeding coefficient representing the whole genome (F ELA‐H); model AICc value was equal to 5368. [file AGE-52-422-s001.docx]

**Supporting Information**

Additional supporting information may be found online in the Supporting Information section at the end of the article.

Table S1. Estimated environmental and inbreeding effects with significant impact on insect bite hypersensitivity prevalence in Old Kladruber Horse: model is based on pedigree relationship matrix and five generations inbreeding coefficient (F_X5_); model AICc value was equal to 5541.

| Effects | Classes | Estimate | Standard error | Odds ratio | DF | t-value | P> \|t\| |
| --- | --- | --- | --- | --- | --- | --- | --- |
| Year | 1 | -2.8988 | 0.2117 | 0.0551 | 332 | -13.69 | <.0001 |
| class | 2 | -0.7735 | 0.1219 | 0.4614 | 332 | -6.35 | <.0001 |
|  | 3 | -0.8723 | 0.1163 | 0.4180 | 332 | -7.5 | <.0001 |
|  | 4 | -0.5808 | 0.0972 | 0.5595 | 332 | -5.98 | <.0001 |
|  | 5 | -0.3405 | 0.1034 | 0.7114 | 332 | -3.29 | 0.0011 |
| Age | 1-2 | -0.4613 | 0.0984 | 0.6305 | 185 | -4.69 | <.0001 |
|  | 3-5 | -0.9700 | 0.0915 | 0.3791 | 185 | -10.61 | <.0001 |
|  | >5 | -1.8483 | 0.1378 | 0.1575 | 185 | -13.42 | <.0001 |
| Sex | Males | -1.2855 | 0.1151 | 0.2765 | 555 | -11.17 | <.0001 |
|  | Females | -0.9009 | 0.1016 | 0.4062 | 555 | -8.87 | <.0001 |
| Inbreeding | F_X5_ | 0.0714 | 0.0253 | 1.0740 | 555 | 2.82 | 0.0049 |

Table S2. Estimated environmental and inbreeding effects with significant impact on insect bite hypersensitivity prevalence in Old Kladruber Horse: model is based on pedigree relationship matrix and classical inbreeding coefficient (F_X_); model AICc value was equal to 5546.

| Effects | Classes | Estimate | Standard error | Odds ratio | DF | t-value | P> \|t\| |
| --- | --- | --- | --- | --- | --- | --- | --- |
| Year | 1 | -2.9198 | 0.2125 | 0.0539 | 332 | -13.74 | <.0001 |
| class | 2 | -0.8000 | 0.1227 | 0.4493 | 332 | -6.52 | <.0001 |
|  | 3 | -0.8732 | 0.1168 | 0.4176 | 332 | -7.48 | <.0001 |
|  | 4 | -0.5830 | 0.0975 | 0.5582 | 332 | -5.98 | <.0001 |
|  | 5 | -0.3300 | 0.1032 | 0.7189 | 332 | -3.2 | 0.0015 |
| Age | 1-2 | -0.4857 | 0.0989 | 0.6153 | 185 | -4.91 | <.0001 |
|  | 3-5 | -0.9812 | 0.0919 | 0.3749 | 185 | -10.68 | <.0001 |
|  | >5 | -1.8368 | 0.1379 | 0.1593 | 185 | -13.32 | <.0001 |
| Sex | Males | -1.2909 | 0.1154 | 0.2750 | 555 | -11.18 | <.0001 |
|  | Females | -0.9115 | 0.1017 | 0.4019 | 555 | -8.96 | <.0001 |
| Inbreeding | F_X_ | 0.0424 | 0.0192 | 1.0434 | 555 | 2.21 | 0.0277 |

Table S3. Estimated environmental and inbreeding effects with significant impact on insect bite hypersensitivity prevalence in Old Kladruber Horse: models is based on pedigree relationship matrix and Kalinowski’s new inbreeding coefficient (F_NEW_); model AICc value was equal to 5551.

| Effects | Classes | Estimate | Standard error | Odds ratio | DF | t-value | P> \|t\| |
| --- | --- | --- | --- | --- | --- | --- | --- |
| Year | 1 | -2.9422 | 0.2140 | 0.0527 | 332 | -13.75 | <.0001 |
| class | 2 | -0.7865 | 0.1224 | 0.4554 | 332 | -6.43 | <.0001 |
|  | 3 | -0.8682 | 0.1167 | 0.4197 | 332 | -7.44 | <.0001 |
|  | 4 | -0.5810 | 0.0974 | 0.5593 | 332 | -5.96 | <.0001 |
|  | 5 | -0.3329 | 0.1035 | 0.7168 | 332 | -3.22 | 0.0014 |
| Age | 1-2 | -0.4759 | 0.0987 | 0.6213 | 185 | -4.82 | <.0001 |
|  | 3-5 | -0.9785 | 0.0920 | 0.3759 | 185 | -10.64 | <.0001 |
|  | >5 | -1.8521 | 0.1385 | 0.1569 | 185 | -13.37 | <.0001 |
| Sex | Males | -1.2896 | 0.1156 | 0.2754 | 555 | -11.15 | <.0001 |
|  | Females | -0.9147 | 0.1020 | 0.4006 | 555 | -8.97 | <.0001 |
| Inbreeding | F_NEW_ | 0.1378 | 0.0707 | 1.1477 | 555 | 1.95 | 0.0516 |

Table S4. Estimated environmental and inbreeding effects with significant impact on insect bite hypersensitivity prevalence in Old Kladruber Horse: models is based on single-step relationship matrix and inbreeding coefficient representing the *ELA class II* region (F_ELA-H_); model AICc value was equal to 5319.

| Effects | Classes | Estimate | Standard error | Odds ratio | DF | t-value | P> \|t\| |
| --- | --- | --- | --- | --- | --- | --- | --- |
| Year | 1 | -2.4051 | 0.1804 | 0.0903 | 332 | -13.33 | <.0001 |
| class | 2 | -1.0499 | 0.1274 | 0.3500 | 332 | -8.24 | <.0001 |
|  | 3 | -1.0519 | 0.1191 | 0.3493 | 332 | -8.83 | <.0001 |
|  | 4 | -0.7633 | 0.1031 | 0.4661 | 332 | -7.4 | <.0001 |
|  | 5 | -0.5169 | 0.1063 | 0.5964 | 332 | -4.86 | <.0001 |
| Age | 1-2 | -0.6424 | 0.1016 | 0.5260 | 185 | -6.33 | <.0001 |
|  | 3-5 | -1.0659 | 0.0975 | 0.3444 | 185 | -10.93 | <.0001 |
|  | >5 | -1.7640 | 0.1396 | 0.1714 | 185 | -12.63 | <.0001 |
| Sex | Males | -1.3315 | 0.1309 | 0.2641 | 555 | -10.17 | <.0001 |
|  | Females | -0.9834 | 0.1177 | 0.3740 | 555 | -8.36 | <.0001 |
| Inbreeding | F_ELA-H_ | 0.0180 | 0.0081 | 1.0181 | 555 | 2.22 | 0.0270 |

Table S5. Estimated environmental and inbreeding effects with significant impact on insect bite hypersensitivity prevalence in Old Kladruber Horse: model is based on single-step relationship matrix and inbreeding coefficients representing the *ELA class II* region (*F*_noELA-H_) and the whole genome without the *ELA class II* region (*F*_noELA-H_); model AICc value was equal to 5361.

| Effects | Classes | Estimate | Standard error | Odds ratio | DF | t-value | P> \|t\| |
| --- | --- | --- | --- | --- | --- | --- | --- |
| Year | 1 | -2.4176 | 0.1829 | 0.0891 | 332 | -13.22 | <.0001 |
| class | 2 | -1.0847 | 0.1298 | 0.3380 | 332 | -8.36 | <.0001 |
|  | 3 | -1.0812 | 0.1211 | 0.3392 | 332 | -8.93 | <.0001 |
|  | 4 | -0.7846 | 0.1050 | 0.4563 | 332 | -7.47 | <.0001 |
|  | 5 | -0.5449 | 0.1083 | 0.5799 | 332 | -5.03 | <.0001 |
| Age | 1-2 | -0.6687 | 0.1033 | 0.5124 | 185 | -6.47 | <.0001 |
|  | 3-5 | -1.0939 | 0.0993 | 0.3349 | 185 | -11.02 | <.0001 |
|  | >5 | -1.7853 | 0.1423 | 0.1677 | 185 | -12.54 | <.0001 |
| Sex | Males | -1.3661 | 0.1335 | 0.2551 | 554 | -10.24 | <.0001 |
|  | Females | -0.9991 | 0.1189 | 0.3682 | 554 | -8.40 | <.0001 |
| Inbreeding | F_ELA-H_ | 0.0155 | 0.0083 | 1.0156 | 554 | 1.87 | 0.0620 |
|  | F_noELA-H_ | 0.0614 | 0.0293 | 1.0634 | 554 | 2.10 | 0.0365 |

Table S6. Estimated environmental and inbreeding effects with significant impact on insect bite hypersensitivity prevalence in Old Kladruber Horse: model is based on single-step relationship matrix and inbreeding coefficient representing the whole genome (F_ELA-H_); model AICc value was equal to 5368.

| Effects | Classes | Estimate | Standard error | Odds ratio | DF | t-value | P> \|t\| |
| --- | --- | --- | --- | --- | --- | --- | --- |
| Year | 1 | -2.4051 | 0.1804 | 0.0903 | 332 | -13.33 | <.0001 |
| class | 2 | -1.0499 | 0.1274 | 0.3500 | 332 | -8.24 | <.0001 |
|  | 3 | -1.0519 | 0.1191 | 0.3493 | 332 | -8.83 | <.0001 |
|  | 4 | -0.7633 | 0.1031 | 0.4661 | 332 | -7.4 | <.0001 |
|  | 5 | -0.5169 | 0.1063 | 0.5964 | 332 | -4.86 | <.0001 |
| Age | 1-2 | -0.6424 | 0.1016 | 0.5260 | 185 | -6.33 | <.0001 |
|  | 3-5 | -1.0659 | 0.0975 | 0.3444 | 185 | -10.93 | <.0001 |
|  | >5 | -1.7640 | 0.1396 | 0.1714 | 185 | -12.63 | <.0001 |
| Sex | Males | -1.3315 | 0.1309 | 0.2641 | 555 | -10.17 | <.0001 |
|  | Females | -0.9834 | 0.1177 | 0.3740 | 555 | -8.36 | <.0001 |
| Inbreeding | F_ELA-H_ | 0.0180 | 0.0081 | 1.0181 | 555 | 2.22 | 0.0270 |
